# Cigarette Smoking Increases Abdominal and Visceral Obesity but Not Overall Fatness: An Observational Study

**DOI:** 10.1371/journal.pone.0045815

**Published:** 2012-09-24

**Authors:** Jung Hwan Kim, Kyung Won Shim, Yeong Sook Yoon, Sang Yeoup Lee, Sung Soo Kim, Sang Woo Oh

**Affiliations:** 1 Department of Family Medicine, Seoul Eulji Hospital, Eulji University, Seoul, South Korea; 2 Department of Family Medicine, Ewha Woman’s University Mokdong Hospital, Seoul, South Korea; 3 Department of Family Medicine, Center for Health Promotion, and Clinical Research Center, Ilsan-paik Hospital, Inje University, College of Medicine, Gyeonggi-Do, South Korea; 4 Department of Family Medicine, Pusan National University, College of Medicine, Busan, South Korea; 5 Department of Family Medicine, Chungnam National University Hospital, Daejeon, South Korea; 6 Department of Family Medicine, Center for Obesity, Metabolism, and Clinical Nutrition, Dongguk University International Hospital, Gyeonggi-Do, South Korea; Brigham and Women’s Hospital and Harvard Medical School, United States of America

## Abstract

**Background:**

Cigarette smoking and obesity are leading public health concerns. Both increase the risk for cardiovascular disease, cancer, and metabolic abnormalities. This study was conducted to assess the association between cigarette smoking and different types of obesity.

**Methodology/Principal Findings:**

Two hundred eighty-three visitors to university hospitals located in four main provinces of South Korea were participated. All participants were classified as either current/past or never smokers and were divided into quartiles according to the total pack-years. Body mass index, waist circumference, total body fat percentage, and area of visceral and abdominal subcutaneous fat were measured. These results of each groups were compared. Waist circumference, and visceral fat area showed a J- or U-shaped association with total smoking amount during a lifetime. After restricting the analyses to past/current smokers, we found significant dose-dependent associations of smoking pack-years with abdominal and visceral obesity. Overall obesity measured by body mass index and total body fat percentage did not show such associations. Although current smokers clearly showed significant associations, we could not demonstrate these in past smokers, possibly because of the limited sample size.

**Conclusions/Significance:**

Although smokers did not show significant difference in mean body mass index than those who never smoked, they showed more metabolically adverse fat distributions with increasing smoking amounts. This finding suggests that smoking is not beneficial for weight control. Therefore, smoking cessation and avoidance of smoking commencement should be addressed as important public health issues in preventing obesity and related complications.

## Introduction

Cigarette smoking and obesity are leading public health concerns facing modern societies. Both increase the risk for cardiovascular disease [1], cancer [2], and metabolic abnormalities [3], although the mechanisms by which they do so appear to differ.

It has been well established that smokers generally have a lower body mass index (BMI) than non-smokers and that smoking cessation is often associated with an increase in body weight [4], [5]. For many people, the fear of weight gain is a considerable barrier to smoking cessation [6]. However, several recent studies have suggested that cigarette smoking increases abdominal obesity [7]–[12].As abdominal obesity is a greater risk factor than overall obesity for obesity-related comorbidities and mortality, this finding suggests an important clinical need to discourage smoking as a method of weight control.

Previous studies on abdominal obesity have focused predominantly on measures of waist circumference (WC) or the waist-to-hip ratio (WHR) [7]–[12]. However, the impacts of visceral and subcutaneous abdominal fat may differ, and thus the question of whether smoking increases abdominal obesity by increasing visceral adiposity or subcutaneous adiposity warrants investigation [13]. Another issue that merits clarification is whether the association between smoking and abdominal obesity merely reflects differences in lifestyle factors, such as physical activity and alcohol consumption. Given the cumulative effects of smoking, we evaluated the combined effects of the total amount of smoking during a lifetime (pack-years). To augment our understanding of the association between smoking and abdominal fatness, we used a relatively large amount of data obtained via computed tomography (CT) and others.

## Methods

### Ethics Statement

This study was approved by the Institutional Review Board (IRB) of Ilsan-paik Hospital. All subjects provided written informed consent to participate in the study.

### Subjects and Measurements

Data of 283 men who visited to university hospitals located in four main provinces of Korea, from 1 September to 31 December 2004, were evaluated. The subjects’ medical histories were carefully ascertained by nurses or physicians and included questions regarding age, education level, occupation, income, marital status, smoking habits, alcohol consumption, exercise, previous and current diseases, and family history of diseases. Subjects who exercised regularly with moderate intensity were questioned regarding the frequency at which they exercised per week and the length of time per exercise session. Alcohol consumption was assessed by questioning subjects regarding their drinking behaviour during the month prior to the medical evaluation.

The degree of overall and abdominal obesity was assessed using the body mass index and waist circumference. Height and body weight were measured using a digital scale, with the subject wearing a light gown. Waist circumference was measured to the nearest 0.1 cm with a tape measure at the midpoint between the lower costal margin and the iliac crest. Total body fat percentage (BF%) was assessed using a eight-polar tactile-electrode impedance-meter(Inbody 3.0,Biospace, Seoul, Korea) [14]. The subjects were asked not to eat or drink for 2 hours prior to the physical examination. Abdominal fat was assessed from the CT scans taken at the L4–L5 level. Abdominal fat was defined as the area corresponding to the pixel range from −190 to −30 Hounsfield units [15].The areas of visceral and subcutaneous abdominal adipose tissue were measured. The fat inside the peritoneum was considered visceral adipose tissue, and the fat between the dermis and muscle fascia was considered subcutaneous adipose tissue.

Overall obesity was defined as a BMI ≥25 kg/m^2^, as recommended by the World Health Organizationwestern pacific regions and others [1]. Overall body fatness was defined by BF% of >25% in men [16]. Abdominal obesity was defined as a WC>90 cm [1]. As well-established cut-off points for visceral fat areas were unavailable, previously suggested cut-off points of 100 and 130 cm^2^ were used to define visceral obesity [17], [18].

Smoking status was divided into three categories: past, current, and never. Past smokers were defined as those who had abstained from smoking for more than 3 months at the time of the evaluation. The total pack-years were calculated from the total number of years spent smoking multiplied by the number of packs smoked daily. However, a clear classification of total pack-years during a lifetime could not be determined. Therefore, the total pack-years of all past/current smokers were divided into quartiles for detailed analyses, such as dose-dependent associations.

### Statistical Analyses

The study subjects were classified as either current/past or never smokers. The total pack-years were divided into quartiles. One-way analysis of variance (ANOVA) followed by Duncan’s post-hoc test was used to determine the significance of the differences among the mean values of these groups. Polynomial contrasts were applied to test for linear and quadratic trend. Multiple logistic regression analysis was used to calculate the odds ratios for overall, abdominal, and visceral obesity. A model adjusted for age (10-year categories), exercise frequency (none, 1–2, 3–4, and ≥5 times per week with duration of ≥30 min), and alcohol consumption (continuous)was constructed. The linear trend in the odds ratio was evaluated using the trend test. Separate analyses according to current or past smoking status were additionally conducted to identify any associations. All analyses were two-tailed, with significance chosen as P<0.05. All statistical analyses were conducted using SPSS, version 11.0.1,for Windows (SPSS Inc., Chicago, IL, USA).

## Results

The study subjects had an average BMI of 26.4±4.8 kg/m^2^ and an average WC of 89.0±12.4 cm. The mean areas of visceral and abdominal subcutaneous fat were 118.7±62.9 and 187.8±10.7 cm^2^, respectively. However, most other anthropometric measures did not show any statistical differences between never- and past/current smoker groups (Table 1). Only average daily alcohol consumption amount show difference significantly among each groups.

**Table 1:** Clinical Characteristics of Study Subjects according to Smoking Status.

Characteristics	Never smokers	Past smokers	Current smokers
Number (persons)	105	60	118
Age (years)	38.1±9.3	39.4±9.0	36.3±7.5
Weight (kg)	79.7±16.4	76.6±10.4	76.9±16.6
Height (cm)	171.1±7.0	172.0±6.0	172.2±7.7
Body Mass Index	27.2±5.1	25.9±3.3	25.9±5.2
Waist circumference (cm)	90.6±12.9	88.6±9.5	87.7±13.3
Visceral fat (cm2)	125.4±66.4	118.9±58.3	112.6±61.7
Subcutaneous abdominal fat (cm2)	195.2±104.7	173.8±70.3	188.4±109.9
Average daily alcohol consumption amount (cal/week)†	98.8±118.4	181.0±159.9	165.4±121.7
Exercise frequencies per week (%)			
1∼2	8(7.6)	3 (5.0)	6 (5.1)
3∼4	3(2.9)	1 (1.7)	4 (3.4)
≥5	3(2.9)	5 (8.3)	3 (2.5)

Data are presented as means ± SD.

* P value <0.05,

† P value<0.001.

All anthropometric measures did not show linear trends (Table 2). However, anthropometric measures such as BMI (P = 0.005), WC (P = 0.001), and visceral fat area (P<0.001), showed quadratic trends (U- or J-shaped association) with increasing total smoking amounts. BF% and abdominal subcutaneous fat did not show such significant associations.

**Table 2:** Comparing Mean Values of Anthropometric Measures with Smoking Amounts during a Lifetime.

	Smoking Amount					P for combined	P for linear	P for quadratic
	Never(105)	Q1 (44)	Q2(44)	Q3(46)	Q4(44)			
Smoking amount (Pack-years)	0^a^	4.1±1.9^b^	9.4±0.9^ c^	16.0±2.4^d^	27.8±8.2^e^	<0.001	<0.001	<0.001
Height (cm)	171.1±7.0^a^	172.0±8.3^a,b^	172.1±7.0^a,b^	174.2±6.4^b^	170.1±6.5^a^	0.065	0.608	0.036
Weight (kg)	79.7±16.4	77.9±20.2	74.3±12.7	76.1±10.3	78.6±14.3	0.326	0.328	0.077
BMI (kg/m^2^)	27.2±5.1^b^	26.3±6.1^a,b^	25.1±4.1^a^	25.1±3.4^a^	27.2±4.4^b^	0.031	0.220	0.005
BF%	25.9±7.1	26.3±9.2	23.1±6.0	24.0±6.2	25.9±6.9	0.120	0.301	0.079
WC (cm)	90.6±12.9^b^	88.5±15.7^a,b^	83.8±9.4^a^	87.5±9.6^a,b^	92.1±11.6^b^	0.012	0.718	0.001
Visceral fat (cm^2^)	125.4±66.4^b,c^	92.6±47.1^a^	104.8±54.8^a,b^	118.0±57.9^b^	143.4±69.8^c^	0.001	0.223	<0.001
SCF(cm^2^)	195.2±104.7^a,b^	219.1±147.0^b^	169.2±78.2^a^	168.2±62.0^a^	182.7±78.4^a,b^	0.059	0.076	0.961

BMI = Body Mass Index, BF% = Body Fat Percentage, WC = Waist circumference, SCF = Abdominal Subcutaneous Fat.

Means in the same row with different superscript letters are significantly different, P<0.05 (ANOVA with Duncan’s post hoc test).

The lacks of association were also observed in the logistic regression analyses (Table 3). However, when the analyses were confined to past/current smokers, significant dose-dependent associations were observed with abdominal obesity defined by WC>90 cm (P = 0.004) and visceral fatness defined by visceral fat area>100 cm^2^ (P = 0.012) or 130 cm^2^ (P = 0.016). But we could not demonstrate it with overall obesity (BMI ≥25 kg/m^2^) and overall body fatness (by BF% ≥25%). The associations with abdominal obesity (P = 0.006) and visceral fatness (P = 0.019 and 0.020) were still observed when the analyses were restricted to current smokers (n = 118), but only abdominal obesity (P = 0.024) when restricted to past smokers (n = 60, Table 4).

**Table 3:** Adjusted Odds Ratio of Visceral Obesity and Others according to the Smoking Amounts during a Lifetime.

	All subjects						Past and Current smokers				
	Smoking Amount (Pack-years)						Smoking Amount (Pack-years)				
	Never(105)	Q1 (44)	Q2(44)	Q3(46)	Q4(44)	P fortrend	Q1(44)	Q2 (44)	Q3 (46)	Q4 (44)	P for trend
BMI≥25 kg/m^2^	Ref	0.79 (0.38–1.68)	0.53 (0.25–1.14)	0.58 (0.29–1.16)	1.07 (0.41–2.80)	0.276	Ref	0.61 (0.25–1.45)	0.86 (0.37–1.99)	1.84 (0.58–5.83)	0.520
BF% ≥25%	Ref	0.55 (0.26–1.20)	0.33 (0.15–0.78)	0.44 (0.22–0.92)	1.45 (0.57–3.66)	0.276	Ref	0.58 (0.22–1.51)	0.91 (0.37–2.27)	4.27 (1.24–14.71)	0.081
WC>90 cm	Ref	0.34 (0.15–0.75)	0.30 (0.13–0.69)	0.62 (0.31–1.25)	1.34 (0.53–3.42)	0.552	Ref	0.77 (0.28–2.11)	2.12 (0.84–5.30)	5.33(1.61–17.63)	0.004
Visceral fat>100 cm^2^	Ref	0.36 (0.16–0.78)	0.77 (0.36–1.62)	0.92 (0.46–1.85)	0.89 (0.34–2.30)	0.990	Ref	2.03 (0.84–4.95)	2.87 (1.21–6.84)	3.29(1.02–10.65)	0.012
Visceral fat>130 cm^2^	Ref	0.40 (0.17–0.94)	0.51 (0.22–1.19)	0.85 (0.42–1.72)	1.24 (0.50–3.06)	0.947	Ref	1.25 (0.44–3.57)	2.19 (0.85–5.69)	3.96(1.18–13.21)	0.016

BMI = Body Mass Index, BF% = Body Fat Percentage, WC = Waist Circumference.

**Table 4:** Adjusted Odds Ratio of Visceral Obesity and Others in Current and Past smokers.

	Current smokers						Past smokers			
	Smoking Amount (Pack-years)						Smoking Amount (Pack-years)			
	Q1 (33)	Q2 (25)	Q3 (30)	Q4 (30)	P for trend	Q1 (11)	Q2 (19)	Q3 (16)	Q4 (14)	P for trend
BMI≥25 kg/m^2^	Ref	0.44 (0.14–1.39)	0.89 (0.32–2.46)	3.55 (0.74–17.15)	0.285	Ref	1.29 (0.25–6.60)	0.86 (0.17–4.42)	1.41 (0.15–13.34)	0.925
BF% ≥25%	Ref	0.19 (0.05–0.74)	0.57 (0.18–1.78)	3.67 (0.71–19.02)	0.393	Ref	5.31 (0.70–40.25)	3.21 (0.43–24.19)	8.49 (0.78–92.62)	0.114
WC>90 cm	Ref	0.60 (0.15–2.47)	2.32 (0.74–7.28)	8.41 (1.71–41.37)	0.006	Ref	2.07 (0.27–16.05)	4.04 (0.55–29.80)	14.47 (1.11–188.05)	0.024
Visceral fat>100 cm^2^	Ref	1.85 (0.60–5.74)	4.04 (1.41–11.58)	2.79 (0.64–12.20)	0.019	Ref	4.34 (0.72–26.06)	1.50 (0.27–8.36)	8.55 (0.70–103.91)	0.223
Visceral fat>130 cm^2^	Ref	1.69 (0.44–6.46)	2.86 (0.87–9.40)	5.52 (1.14–26.81)	0.020	Ref	1.62 (0.22–11.97)	2.06 (0.32–13.32)	5.17 (0.52–51.06)	0.163

BMI = Body Mass Index, BF% = Body Fat Percentage, WC = Waist Circumference.

## Discussion

The present study, which controlled for age, alcohol consumption, and exercise, demonstrated that smoking increases abdominal and visceral fatness in smokers. We also found that the positive association of abdominal obesity with smoking is primarily mediated by an increase in visceral fat.

This study had inherent limitations. The cross-sectional observational design did not allow us to establish any definitive temporal associations for identifying clear causal relationships. We relied on self-reported measures of smoking habits, and we did not measure the potential amount of passive smoke exposure in those who never smoked. We could not demonstrate the association for women because their lower smoking rate (only 3.6% of 363 women) limited the ability to detect modest to moderate associations between smoking and obesity. In addition, the study subjects were not a representative sample. However, the strengths of our study included the relatively detailed smoking histories of the subjects and the real measures of visceral and subcutaneous fat obtained via computed tomography scan.

The relationship between adipose tissues and various physiological and pathological processes differs according to the regional adipose depot. Visceral fat is considered a more reliable marker for obesity-related complications than are other anthropometric measures. A higher flux rate of adipose-driven free fatty acid to the liver through the portal vein has been identified as the reason visceral adipose tissue is associated with greater health risks than subcutaneous adipose tissue [13]. They cause hepatic insulin resistance and an increased production of both glucose and very low density lipoprotein by the liver.In the present study, we found that smoking had a positive association with visceral adiposity, whereas an association with subcutaneous fat was not observed.

These findings suggest that smoking can increase obesity-related comorbidities related to increased visceral adiposity. In another study, we reported that two components of metabolic syndrome, elevated triglycerides and low high-density lipoprotein cholesterol levels, were associated with smoking [3]. Consistent with the present study, we also demonstrated an association between smoking and abdominal obesity in past/current smokers. Other researchers demonstrated associations between smoking and other obesity–related comorbidities, such as type 2 diabetes [19], hypertension [20], and insulin resistance [21]. Although the precise mechanism underlying these associations is not fully understood, our findings suggest the possibility that the associations between smoking and various metabolic complications may be mediated an increase in visceral obesity.

Some previous studies show negatively correlation of obesity with smoking [22], [23]. They concluded that current smokers had less BMI or less waist circumference. We found that BMI, waist circumference and visceral fat area were quadratic pattern according to smoking amount. It means that although BMI, waist circumference and visceral fat area were high in never smokers, they would increase with increasing of smoking amount.

Although the effects of smoking are cumulative, the measurements of the amount of smoking in previous studies have generally been limited to the daily number of cigarettes smoked. In the present study, we evaluated the effect of total pack-years during a lifetime, considering both the amount and duration of smoking. Our findings suggest that the cumulative effects of smoking increase body fat, according to waist circumference and measures of visceral fat. However, we did not observe the association of visceral adiposity in past smokers. In past smokers, the period of smoking cessation is an important determinant of abdominal obesity [7]. Some investigators suggested that a long period of smoking cessation tends to reduce abdominal obesity to a level similar to that of those who never smoked and that those who have smoked for longer periods or more heavily require longer periods of smoking cessation to achieve the same result [7]. This observation suggested that the period of smoking cessation needed to be considered in our analyses. However, we could not observe the effects of smoking cessation periods. The reason for failing to find the association of visceral fat with smoking amount in past smoker is not clear, but it may be attributable to the small sample size of the past smokers or to errors in obtaining precise information about smoking cessation periods. Further studies on whether the smoking amounts of past smokers are related to visceral fatness and whether cessation periods affect this association are warranted.

Previous reports have suggested that the aging process increases visceral adiposity [24], physical activity decreases visceral adiposity [25] and alcohol consumption increases abdominal obesity [26]. Even after adjusting for these influences, we were able to demonstrate significant associations between smoking and body fatness. However, the precise biological mechanisms that underlie these associations are difficult to identify because cigarettes contains thousands of compounds and the regulation of body fat is a complex physiological process. As mentioned above, we conducted our study only in men due to low smoking rate in women. Previous study revealed the gender difference of smoking effect on obesity and metabolic parameters [27]. The anti-estrogenic effects of smoking and an imbalance in androgenic and estrogenic activities have been implicated in the mechanism [7], [28], but further studies are required.

In conclusion, although smokers have a lower mean BMI compared with never-smokers, heavier smokers have a more metabolically adverse fat distribution profile, with higher abdominal and visceral adiposity than lighter ones. This observation reflects the metabolic consequences of smoking. Given the association between smoking and visceral fatness, it is clear that smoking cessation and avoidance of smoking commencement should be emphasized in the prevention of obesity and related complications.
